# Experiences with online consultation systems in primary care: case study of one early adopter site

**DOI:** 10.3399/bjgp17X693137

**Published:** 2017-10-10

**Authors:** Michael Casey, Sara Shaw, Deborah Swinglehurst

**Affiliations:** The Chrisp Street Health Centre, London.; Nuffield Department of Primary Care Health Sciences, University of Oxford, Oxford.; Centre for Primary Care and Public Health, Blizard Institute, Queen Mary University of London, London.

**Keywords:** general practice, health services accessibility, physician–patient relations, remote consultation, technological innovations

## Abstract

**Background:**

There is a strong policy drive towards implementing alternatives to face-to-face consultations in general practice to improve access, efficiency, and cost-effectiveness. These alternatives embrace novel technologies that are assumed to offer potential to improve care.

**Aim:**

To explore the introduction of one online consultation system (Tele-Doc) and how it shapes working practices.

**Design and setting:**

Mixed methods case study in an inner-city general practice.

**Method:**

The study was conducted through interviews with IT developers, clinicians, and administrative staff, and scrutiny of documents, websites, and demonstrator versions of Tele-Doc, followed by thematic analysis and discourse analysis.

**Results:**

Three interrelated themes were identified: online consultation systems as innovation, managing the ‘messiness’ of general practice consultations, and redistribution of the work of general practice. These themes raise timely questions about what it means to consult in contemporary general practice. Uptake of Tele-Doc by patients was low. Much of the work of the consultation was redistributed to patients and administrators, sometimes causing misunderstandings. The ‘messiness’ of consultations was hard to eliminate. In-house training focused on the technical application rather than associated transformations to practice work that were not anticipated. GPs welcomed varied modes of consulting, but the aspiration of improved efficiency was not realised in practice.

**Conclusion:**

Tele-Doc offers a new kind of consultation that is still being worked out in practice. It may offer convenience for patients with discrete, single problems, and a welcome variation to GPs’ workload. Tele-Doc’s potential for addressing more complex problems and achieving efficiency is less clear, and its adoption may involve unforeseeable consequences.

## INTRODUCTION

Primary care in England is *‘reaching saturation point’*.^1^ Between 2007 and 2014 the number of consultations with GPs increased by 16%.^1^ In 2015–2016, 12% of GP training posts were unfilled.^2^ In the next 5 years, one-third of GPs plan to retire, and 28% plan to become part-time.^3^ These challenges have prompted calls for alternative models of care, revised skill mix, digital technologies, and increased supported self-management.^4^^–^8

Several technologies provide alternatives to face-to-face consultations. Telephone consulting is well established (66% of practices in England and Scotland report using this).^9^ Although 25% of GPs have exchanged emails with patients, it is not routine practice.^10^ Only 6% of UK practices report using email regularly. Most have no plans to do so.^9^ A pan-European study of email consulting found wide variation in use across countries.^11^

There is concern among GPs that alternatives to face-to-face consultations may increase workload and compromise safety.^3^^,^^9^^,^^12^^,^^13^ The ESTEEM trial of telephone consulting found a 29% reduction of face-to-face contacts over 28 days, but an overall increase (38%) in all contacts.^14^ A Cochrane review of email consultations was inconclusive regarding effect on workload.^15^

Two models of online consultations (also called e-consultations) are currently available.^16^ One is pharmacy led and explicitly avoids contact with the GP (patients obtain private prescriptions for a limited range of conditions online). The other involves web-based history-taking communicated to the patient’s GP surgery, with potential for a face-to-face consultation depending on how the GP interprets the information. Despite equivocal evidence, NHS England plans to offer every practice support to adopt online consultation systems, committing an estimated £45 million investment.^5^

In this article, the authors report a case study of an online consultation system recently incorporated into an inner-city general practice and consider how the introduction of an online consultation system is changing the work of general practice.

## METHOD

The authors conducted a qualitative case study of the development and implementation of an online consultation system (Tele-Doc) by a large, multi-site NHS GP partnership (Forest Group) and linked practice (Willow Surgery). (Tele-Doc, Forest Group, and Willow Surgery are all pseudonyms; see Appendix 1 for further details of Tele-Doc.) The authors conceptualised ‘the case’ as context dependent, and evolving over time.^17^^,^^18^ They collected data from multiple sources, including narrative interviews with a maximum variety sample of seven stakeholders (three development/operational staff at Forest Group, and four end-users, two of whom were GPs and two administrators at Willow Surgery), a purposive sample of six documents (including a pilot report, training presentations, and reports on user demographics), and a review of Tele-Doc.

**How this fits in**

Online consultation systems are proposed as one policy response to increasing workload in primary care. Little is known about how online consultations play out in practice, or what this new consultation is. Structured online consultations may not reduce (and may even increase) overall workload, and may be ill-suited to consulting about complex problems. Expectations that new technologies will increase efficiency may be effective in attracting funding for technology development, but efficiencies may be difficult to achieve in practice.

The authors invited participants to consider an example of using Tele-Doc prior to interview. Interviews lasted up to 50 minutes, used a topic guide (with four narrative-eliciting questions; see Box 1), and enabled insights into how participants construct meaning and identity.^19^^,^^20^

Box 1. **Topic guide and interview questions**

**Topic guide**
Technical evolution of Tele-Doc
Changes to work patterns (individual, organisational)
Training/staff support
Barriers and facilitators to using Tele-Doc
Doctor–patient relationship
Contextual factors influencing Tele-Doc
Patient experience and use of Tele-Doc
Future development of Tele-Doc

**Guiding questions for interview**
1. Could you talk me through your own story of involvement with Tele-Doc?
2. Could you talk me through the example [of use of Tele-Doc] that I asked you to think of before the interview?
3. How, if at all, has Tele-Doc shaped your working practice?
  — Practice/organisational work
4. How, if at all, do you think patient experience is altered by Tele-Doc?
5. Is there something else you would like to talk about?
6. Is there someone else you think we should talk to as part of this project?

Interviews were transcribed and analysed along with institutional documents using thematic analysis (identifying key themes) and then discourse analysis (to understand how and where meanings are constructed),^21^^,^^22^ ‘zooming in’ on the nuance of talk and ‘zooming out’ to broader context in ways that kept interest in actual practice in the foreground.^23^ The authors imported social theory to extend analysis, adopting a sociotechnical orientation^24^^,^^25^ that conceptualises people, technologies, and material artefacts as interconnected networks,^25^^,^^26^ and drawing the focus on to what happened when Tele-Doc was put to use in general practice. The authors also drew on the sociology of expectations literature.^27^

## RESULTS

Findings are organised in three interrelated themes: online consultation systems as innovation, managing the ‘messiness’ of general practice consultations, and redistribution of the work of general practice.

### Online consultation systems as innovation

‘*The* [Forest Group] *have always tried to innovate. Remote consultation is surely an innovation that’s coming. Let’s try and capture that.’*

(Developer, Forest Group)

*‘The big thing for me at the moment is, you know, there is this whole thing about health is the last industry that needs to move online, OK. Everyone does everything else online — banking, travel, everything. So health needs to follow, and* [Tele-Doc] *is a tool that will facilitate that.’*

(Developer, Forest Group)

These quotes highlight the organisational context in which Tele-Doc was developed, with innovation valued as something to strive towards for its own sake. Sociological theory identifies technological innovation as future oriented, framed as a means to develop previously non-existent opportunities in which abstract expectations about the innovation (in this case Tele-Doc) can be shared among groups, providing direction and attracting investment.^27^ Participants’ accounts revealed a sense of the inevitable (*‘an innovation that’s coming’*), with use of future tense (*‘will facilitate’*) aligning with a modernist perspective, in which it is assumed that technology will provide neat solutions to contemporary problems.^28^ Use of extreme case formulations (‘whole’, ‘everyone’, ‘everything’)^29^ and three-part lists (‘banking, travel, everything*’*)^30^ as rhetorical devices inspire confidence that Tele-Doc ‘will facilitate’ increased online health care. The implication is that health could and should ‘move online’, presenting technology as the key driver (rather than, for instance, the needs of patients).

Significant resources were required to develop Tele-Doc:

‘*It was a* [Forest Group] *decision to invest some …* [long pause] *because it is money, in the GP time to assign five GPs, kind of 4 weeks where they didn’t go and do surgeries. They just sat in a room every day and kicked around until they’d kind of refined these templates … that was the kind of tipping point for us, I think. So we then had something like a product … then launched* [Tele-Doc] *with the* [Forest Group] *patients initially*.’

(Developer, Forest Group)

This quote suggests significant organisational slack within Forest Group (essential for development of any innovation),^31^^,^^32^ enabling five GPs to be released from clinical work for 4 weeks to develop Tele-Doc templates, resulting in the emergence of path dependency (‘tipping point’)^27^ when they had ‘something like a product’ to ‘launch’ and expectations became temporarily stabilised.^33^

Expectations about an innovation and its use typically change over time.^27^ Participants described how the implementation of Tele-Doc was an ongoing process, with continuing adjustment of expectations. For example, when Tele-Doc was piloted, GPs were expected to manage three consultations in 10 minutes:

*‘When* [Tele-Doc was] *first introduced, it was a bit of an ask.* [Tele-Doc] *was a pilot scheme in addition to our allocated set number of* [clinics]*. It was roughly 20 contacts per* [clinic]*. We were having three of these per 10 minutes, which was quite … Admittedly you’re expected to do three consults within 10 minutes. And, clearly, that’s not a sustainable ethos, so then you dropped down to two, two consults per 10 minutes, and then, I think, now we are sort of agreed we do one consult per 10 minutes.’*

(Clinician, Willow Surgery)

The difficulty of completing three online consultations in 10 minutes (after a full clinic) is implied (‘*which was quite* …’), leading to a gradual process of reducing to one consultation per 10 minutes. There did not appear to be a clear way forward (*‘I think, now we are sort of agreed …’*), suggesting that Tele-Doc consultations will continue to evolve. Hence, while the original aspiration of developers for increased efficiency helped to attract funding (the pilot was funded by the clinical commissioning group (CCG) and a charity), it did not appear that efficiencies had been gained in practice. Tele-Doc was recast as being of *‘equivalent standing to face-to-face and phone consults’* (pilot report), and a new narrative of ‘respite’ for clinicians during long face-to-face clinics emerged.

The vision for Tele-Doc had further evolved into an ambition to pioneer online consultation systems widely within primary care, offering what developers described as *‘a new channel and a new concept’* that *‘time’s only going to tell on how this does really pan out’*, speculating that it could *‘completely change the model of general practice’.*

### Managing the ‘messiness’ of general practice consultations

‘*I really like the way that* [the Tele-Doc document] *goes through the history and you can scan the yes/no bits quite quickly, and they’re well flagged for bits that you should pay more attention to.*’

(Clinician, Willow Surgery)

Tele-Doc is one expression of a range of contemporary policy and professional developments, including standardisation of care (for example, protocols), a ‘systems’ approach,^34^ aspirations for a 24/7 ‘customer’ service (three participants drew parallels between Tele-Doc and online banking), and diversification of GP roles to include managerial and commercial ventures.^35^ Partners at Forest Group showed considerable flexibility to adapt to this context and embrace its potential for doing things differently.

A key finding was that Tele-Doc embodied a desire on behalf of the developers to make the consultation less ‘messy’ – to *‘carve off 10, 20, 30% of the stuff that comes in general practice, that’s quite easy’* and make it *‘more streamlined’*. These quotes suggest that the ‘easy’ parts of the consultation are readily identifiable and separable from undifferentiated symptoms. General practice consultations have previously been conceptualised as therapeutic in their own right,^36^^–^39 offering patients an opportunity to make sense of their illness by co-constructing narratives in dialogue with their doctor 40^,^^41^ with a clinician who ‘bears witness’ to suffering.^42^^,^^43^ Face-to-face consultations were described as being more ‘taxing’ and involving ‘multiple problems’:

‘Some patients will bring in their child or their partner, or they’ll come in with a second or third problem … that’s where it becomes tiring.’

(Clinician, Willow Surgery)

It was precisely this ‘messiness’ that was difficult to remove from online consultations, with their highly structured organisation. Participants explained that when Tele-Doc was first introduced some patients entered free-text comments into questionnaires designed for conditions unrelated to their own. Patients’ problems did not always fit neatly into the yes/no boxes provided. Forest Group responded:

‘[Developing] *something called the “generic template”, where you go on if you’ve got an undifferentiated symptom … It was dramatic. Within a week twice as many Tele-Docs were coming through*.’

(Developer, Forest Group)

The ‘generic’ template (‘general advice’ in the patient’s view of Tele-Doc) allowed patients to express their concerns. It quickly gained popularity over condition-specific templates. As Morton and Cornwell have argued, the *‘irreducible variability’* implicit in health care presents difficulties when attempts are made to rely on standardising approaches.^34^ They go on to suggest that ‘*the only way to eliminate variability completely would be to eliminate patients*’.^34^ Arguably, the online consultation goes some way towards this, albeit it also offers convenience for patients who do not want to travel or wait. Minimising variability may succeed in reducing the emotional labour of consulting (making it less ‘tiring’). However, patients appreciate free expression. One clinician speculated upon a possible future with:

‘*Tele-Doc automatically recognising that there is nothing here that a GP needs to do.*’

### Redistribution of the work of general practice

*‘“I am doing Tele-Doc.” They* [the other administrators] *all know that means “stay away.*”’

(Administrator, Willow Surgery)

One consequence of Tele-Doc was redistribution of work from GPs to administrators and patients. Patients took on consulting work by completing a ‘very thorough history’, frequently involving many ‘pages’ of questions. New organisational routines were worked out by administrators (for example, sorting incoming Tele-Doc templates to identify appropriate recipients, entering Tele-Doc consultations into appointment slots).

The assumptions underpinning Tele-Doc emphasised standardised working methods (geared towards achieving manageable workloads and efficiency savings) and created new work for administrators (*‘doing Tele-Doc’*) and a shared understanding that staff would not be disturbed when engaged in it. The combination of an extreme case formulation (*‘all’*)^29^ and the voiced imperative (*‘stay away’*) convey the intensity of this work. The need for ‘more attention and focus’ was in part to minimise new errors that were possible since integrating Tele-Doc. For example, if a Tele-Doc consultation was allocated an appointment slot in the clinical system, this would prompt an automated text message to the patient (inviting them to an appointment). To avoid this, administrators developed a ‘workaround’, booking an ‘unregistered’ patient instead, then typing the patient’s name in small font indicating ‘online consultation’.

Administrators received Tele-Doc templates by email, uploaded them as attachments into electronic records (*‘a lot of clicking’*) and decided how to allocate them. This process was prone to misunderstanding and error. For example, it was possible for unregistered patients to submit templates to Willow Surgery. Patients also used Tele-Doc in unintended ways:

‘Some patients use it as a place to complain. They find it is a way to get us to sit up and complain … not about Tele-Doc … generally about the actual practice.’

(Administrator, Willow Surgery)

The practice had received minor complaints via Tele-Doc (for example, about waiting too long for an appointment) that, although unlikely to warrant a formal complaint, nonetheless demanded attention

(*‘they … get us to sit up’*).

Uptake of Tele-Doc at Willow Surgery had been low, with 0–10 templates submitted daily (one GP said, *‘We have so few it hasn’t really made a dent’*). However, the administrative burden was substantial, beginning with reading the completed template to decide whether to ‘book’ an online consultation (meaning allocate it an appointment slot for GP review), and deciding whether work was clinical or administrative. For example, if on reading the Tele-Doc template administrators decided there was *‘nothing a doctor really could do over the phone. The doctor has to see it’* (the example was a rash), they would book a face-to-face consultation, taking on some aspects of clinical decision making themselves.

GPs had complained that they received requests they deemed administrative (*‘Stuff that should be filtered out at reception’*) and had called for careful attention to this classification work. It had not been entirely successful:

*‘If I find I don’t know* [if the Tele-Doc is clinical or administrative work] *then I will approach someone who is higher up than me. I will either talk to the doctor, or I will talk to one of the admin staff.’*

(Administrator, Willow Surgery)

The adaptation of working patterns to the technology, and parallel adaptation of the technology to meet different staff groups’ needs, was not anticipated. Tele-Doc training focused on technical aspects of using the software and was described as ‘superficial’ or ‘a single training session’. None of the participants referred to training about the impact of Tele-Doc on the non-technical aspects of their roles.

Tele-Doc developers were aware that implementing Tele-Doc was not straightforward. Tele-Doc is now accessible to 231 UK practices, although approximately one-sixth of these practices do not use it:

‘We’ve seen practices switch it off … It’s effectively who holds the power in practice, and it’s not always the GPs. It’s maybe the practice manager, for example … they said, “It makes more work for us.”’

(Developer, Forest Group)

The data suggest that Tele-Doc generates substantial work for non-clinical staff who may be important mediators of the success (or not) of technology implementation.

## DISCUSSION

### Summary

Findings show that Tele-Doc represented a small fraction of the clinical work in the case study. However, it constituted a significant re-thinking of what it means ‘to consult’, and aligned with the contemporary impetus for marketisation, standardisation, and commercialisation.^44^ Clinical leads at Forest Group were innovation enthusiasts, viewing technology as a means to improve services, manage demand, and improve efficiency.^45^^,^^46^ However, although the development of Tele-Doc had been successful (in terms of attracting funding, being ‘up and running,’ and dissemination to 231 practices), implementation appeared less successful (uptake by patients remained low; there was little evidence that efficiency gains were realised). This study suggests that clinicians and administrators worked hard to accommodate Tele-Doc, reshaping their working routines accordingly. This involved new work for administrators, different kinds of work for patients, and a need for clinicians to engage in a process of continual adaptation to embed Tele-Doc into clinical practice. The research suggests that — at least for GPs and administrators — the assumed potential of Tele-Doc for increased efficiency is difficult to achieve.

As with other innovations,^47^ further adaptation of Tele-Doc seems likely. Developers and clinicians invested considerable time and resource into Tele-Doc’s initial condition-specific templates. However, it was the ‘generic’ template that proved most popular with patients. This may reflect patients’ reluctance or inability to commit to a specific condition at the outset of their consultation, or a poor fit between the nature of patients’ problems and the algorithmic logic inscribed into Tele-Doc. Further research is needed to explore patients’ experiences of Tele-Doc.

### Strengths and limitations

This study was small, undertaken as an MSc project, and focused on staff not patients. It involved one atypical practice — an early adopter, closely related to the software developers, and interested in commercial opportunity. Participants were likely to be heavily invested in making Tele-Doc work. As case study researchers, the authors prioritise opportunity to learn over concerns about typicality 48 and particularisation over generalisation.^49^ Single case studies, like this one, can be valuable in shaping future research in emerging areas, illuminating matters that typical situations might not, and providing in-depth analysis of what actually happens when (as in this case) technologies are implemented in practice.

### Comparison with existing literature

These findings resonate with existing literature that describes the disappointingly low uptake of many novel technologies in healthcare settings,^50^^–^52 concerns that the push for new technologies is driven by the interests of policy and industry rather than clear evidence of patient benefit,^28^^,^^53^ and the importance of studying technology-in-practice^54^ as transformation, not just implementation.^16^

### Implications for research and practice

These findings challenge some of the assumptions underlying current digital health policy (for example, that technology will save time and money). For instance, although the aspiration that *‘teams* [in general practice] *need support and space if they are to adopt new ways of working’* 5 is welcome, the findings suggest that staff training and support may be insufficient when attempting to introduce new technologies. Ethnography, preferably involving contrasting sites, has potential for illuminating the complexities of introducing online consultation systems.

Empathy, presence, and compassion are traditionally regarded as important hallmarks of good general practice.^55^ Traditionally, being fully ‘present’ involves not only physical co-presence in time/space, but may also include emotional, intellectual, and spiritual presence.^36^ This kind of presence is called into question with the emergence of programmes like Tele-Doc, where patient and clinician are not physically present, rarely in dialogue, and communicating asynchronously. Further work involving both patients and practitioners is needed to fully appreciate the consequences of a shift away from physical presence in consultations, and the implications of online consultation systems for the quality of general practice consultations. For example, how is ‘presence’ negotiated at distance? How is empathy accomplished in alternative modes of consulting? Likewise, the primary care consultation has traditionally been understood to be exception rich, with the GP managing ambiguous and undifferentiated symptoms, tolerating uncertainty,^56^ and bearing the emotional burden that this entails. Tele-Doc may offer convenience for patients with clearly defined problems, and respite from busy clinics for clinicians. However, these findings suggest that the emotional work of consulting may be marginalised, and that there may be important limits about what is achievable in this new genre of consulting.

## Appendix 1. Introduction to the ‘case’

Tele-Doc was developed by the Forest Group in 2012, piloted in 2013, and is used by all Forest Group practices. It was launched as a commercially available product (owned by Forest Group Company, a separate company linked to the Forest Group) in 2014, and is now available for use in 231 UK practices.

Forest Group includes 19 primary care centres (13 GP surgeries and six urgent-care centres, approximately 100 000 registered patients), based in Cityham, a large city with a diverse, high-density population. Willow Surgery is an inner-city surgery with two GP clinical leads, and four salaried GPs, serving a population of 10 000 patients, of whom 10% do not speak English, and 30–40% speak English as a second language.

Remote consultations in Tele-Doc are accessed through the Willow Surgery website. Patients are offered three options — search for specific conditions, ask for general advice (if the patient is not sure of their condition), and request administrative help.

Patients can search >100 common conditions. These are organised by groups, such as breathing problems, women’s health, and mental health, and can also be viewed alphabetically and pictorially by areas of the body. Once a condition is selected, patients can choose self-help information, pharmacy information on relevant over-the-counter medications, direction to the 111 service (a national 24-hour telephone advice service for non-urgent health problems), and a structured online consultation (Tele-Doc). Tele-Doc is available 24 hours a day. Patients can expect a response by the end of the next working day.

A Tele-Doc consultation begins with patients completing a condition-specific online questionnaire (template) that is submitted to the practice as an email. The questionnaire includes five sections, all of which must be completed.

1. About you (for example, age, contact details).
2. Your expectations (free-text boxes, limited to 500 characters).
3. Your condition (systematic questionnaire, with pull-down menus offering tick box answers to multi-level questions on symptoms). Urgent or emergency symptoms prompt the appearance of a red warning box, advising patients to seek urgent/emergency services. If they select ‘End my consultation, I will seek urgent care instead’, the information they have entered is not submitted to the GP practice.
4. Your health (for example, pre-existing conditions).
5. Review and send to GP.

The template is received by the practice as an email attachment and dealt with according to locally specified organisational routines. There is no real-time consultation, no audio or video component, no instant messaging, and no email exchange with the patient.
